# Complete Genome Sequence of a Novel *Dyadobacter* sp. Strain, NIV53, Isolated from 2-Meter Deep Subsurface Sediment

**DOI:** 10.1128/MRA.00754-21

**Published:** 2021-11-04

**Authors:** Frederik Bak, Alexander Pil Henriksen, Tue Kjærgaard Nielsen, Mette Haubjerg Nicolaisen

**Affiliations:** a University of Copenhagen, Department of Plant and Environmental Sciences, Copenhagen, Denmark; University of Southern California

## Abstract

Here, we provide the complete genome sequence of the subsurface bacterial isolate *Dyadobacter* sp. strain NIV53, a candidate species from the *Spirosomaceae* family. The isolate contained one 7,587,604-bp chromosome, with a GC content of 40.4%, and one plasmid, pNIV1, with a size of 12,453 bp.

## ANNOUNCEMENT

The genus *Dyadobacter* contains aerobic species that have been isolated from a range of environments, including surface-sterilized maize stems, deep (>50 m) subsurface, contaminated soil, and Arctic soil (1–6). Species belonging to this genus are aerobic. Most isolated species are able to grow at low temperatures. The putative new species of *Dyadobacter* was isolated from sediment collected from a borehole (depth of 2 to 2.5 m) near Nivaa, Denmark (55.927363N, 12.491562E). At this depth, the organic carbon level is low (total organic carbon level, 0.28%) and the water table fluctuates, resulting in changing osmotic and oxygen conditions for the bacteria colonizing the soil (7).

The pH (CaCl_2_) of the sediment was 5.7. The sediment sample was sieved (2 mm), mixed with 1× phosphate-buffered saline (PBS), shaken, and sonicated before the suspension was filtered (0.8 μm). The suspension was streaked on 1:10 Reasoner's 2A (R2A) agar plates. Yellow colonies appeared after 2 weeks of incubation at 20°C. Subsequently, one colony was reinoculated on 1:10 R2A agar plates and incubated at 20°C. The strain was designated NIV53.

Bacterial DNA was extracted from an overnight culture grown in liquid R2A medium at 20°C using a Qiagen Genomic-tip (20/G) and genomic buffers (Qiagen) and was subjected to Nanopore sequencing. Whole-genome sequencing was performed using a MinION sequencer (Oxford Nanopore Technologies, Oxford, UK). Library preparation was performed using the rapid barcoding sequencing kit (SQK-RBK004), and sequencing was performed on a MinION Mk1B system using an R9.4 flow cell. No size selection was performed prior to library preparation. A total of 109,401 reads were generated, with a total of 362,808,484 bp and an *N*_50_ value of 6,205 bp. Base calling and demultiplexing of barcoded samples was performed using Guppy (v. 5.0.7). Default parameters were used for all software. The adapters were trimmed from the reads using Porechop v. 0.2.4 (https://github.com/rrwick/Porechop), and the resulting sequences were assembled with Flye v. 2.7 (8). The assembly was polished with Medaka v. 1.1.1 (https://nanoporetech.github.io/medaka). This resulted in two circularized scaffolds, corresponding to a chromosome (7,587,604 bp) and a plasmid (12,453 bp), with a total length of 7,600,057 bp and a GC content of 40.4%. The final genome coverage was 53×. The genome was manually inspected and verified to be circular using CLC Genomics Workbench v. 12 (https://digitalinsights.qiagen.com). The genome was annotated using the NCBI Prokaryotic Genome Annotation Pipeline (PGAP) v. 5.2 (9).

PGAP identified 6,199 coding sequences in NIV53, 39 tRNAs, and 3 rRNA operons. Additionally, the genome contained genes encoding catalase peroxidase and superoxide dismutases. A plasmid replication initiation protein-encoding gene was manually identified on the 12-kbp plasmid pNIV1, using online BLASTP. A BLAST search of the 16S rRNA gene sequence against the EzBioCloud 16S rRNA database (10) revealed that *Dyadobacter* sp. strain NIV53 had the greatest similarity to Dyadobacter koreensis (98.2%). The average nucleotide identity (ANI) between strain NIV53 and D. koreensis was 73%, as calculated using the ANI calculator (11). The complete genome sequence of this strain expands the available genomic information for subsurface bacteria, supporting future studies of the ecology of subsurface bacteria.

### Data availability.

The whole-genome shotgun project has been deposited in GenBank with the accession numbers CP081298 and CP081299 under BioProject PRJNA748431. Base-called Nanopore sequence reads are available under SRA accession number SRR15221434.
